# Aortic dissection in a Jehovah’s witness: Frozen Elephant Trunk and subsequent 4D flow MRI analysis, a case report

**DOI:** 10.1186/s13019-026-03889-1

**Published:** 2026-02-19

**Authors:** Laura Asta, Valentina Mancini, Cesare Mantini, Fabrizio Ricci, Umberto Benedetto

**Affiliations:** 1 Department of Neuroscience, Imaging and clinical Sciences, Cardiac Surgery Dept. University “G.d’Annunzio” Chieti-Pescara, Via dei Vestini, 66100 Chieti, Italy; 2 Division of Cardiac Surgery, SS Annunziata Hospital, Via dei Vestini, 66100 Chieti, Italy

**Keywords:** Acute aortic dissection, Aortic aneurysm, Frozen Elephant Trunk, Jehovah’s witness, 4D flow MRI

## Abstract

Thoracic aortic surgery in Jehovah’s Witness (JW) patients presents a challenge to the surgeon due to the patient’s religious-based objection to the use of allogeneic blood products. Recently Frozen elephant trunk (FET) has been proposed as a more definitive treatment for patient presenting with thoracic aortic aneurysm and dissection (TAAAD) especially when the aortic arch is involved. We report a case of treating acute type A aortic dissection with the use of the Frozen Elephant Trunk technique in a 75-year old JW patient. The bloodless strategy included: minimize cardiopulmonary bypass and crossclamp time, minimize hemodilution, the use of intra-operative cell salvage, autologous blood trasfusion and the use of topical hemostatic agents. FET can reduce the risk of bleeding as it protects the distal anastomosis from bleeding by covering it with the stent. To our knowledge this is the fist report of a FET performed in a JW patient with TAAAD. Furthermore, the patient underwent 4D (Four Dimensional) Flow MRI (Magnetic Resonance Imaging) analysis two and a half years after the operation which allowed us to confirm the excellent outcome of the operation and the perfect seal of the prosthesis used.

## Introduction

Bloodless surgery in JW patients is still perceived as a challenge for patients and clinicians due to risk of irreversible severe anaemia. JW patients, in fact, refuse any form of transfusion because they believe that Jehovah will deny eternal salvation to anyone who has received blood from another human being. This refusal is legally recognized as it is based on respect for the individual’s right to privacy and freedom of religious practice [1]. The risk is further increased when a JW patient requires surgery for type A acute aortic dissection which is associated with severe coagulopathy and perioperative bleeding. In fact, the passage of blood into the false lumen in aortic dissection determines an increase in the activation of coagulation factors, fibrinolsis and an increase in platelet activation [2]. Furthermore hypothermia, used as a neuroprotection mechanism in thoracic aortic surgery, causes a further deficit in the enzymatic activation of platelet functions [3]. For this reason, pre, intra and post-operative strategies have been implemented to reduce the risk of bleeding, the need for transfusion and to obtain satisfactory surgical results [4]. To minimize the risk of bleeding in this scenario, a limited repair of the ascending aorta is anticipated to be a reasonable solution. In the last 10 years, FET has become increasingly common as a more definitive treatment for patient presenting with TAAAD especially when the aortic arch is involved with excellent long term outcomes. In particular, FET consists in the use of a hybrid prosthesis made up of a proximal vascular portion in dacron and a distal portion of self-expanding nitinol stent. This prosthetic structure, therefore, allows the treatment of aneurysm and dissection when it affects the ascending thoracic aorta, the aortic arch, and the descending thoracic aorta in a single surgical timing [5]. However, performing FET in JW patients may be perceived as too risky for due to excessive bleeding. To our knowledge this is the fist report of a FET performed in a JW patient with TAAAD. After two and a half years the patient underwent 4D Flow MRI analysis. Time-resolved 3D phase contrast magnetic resonance imaging (4D Flow) is a non-invasive imaging technique, but one that allows for exceptional evaluation of complex flow patterns. This examination allowed us not only to ascertain the stability of the dissecting process, but also to confirm the success of the operation and the excellent performance of the prosthesis used. For this reason, 4D Flow MRI analysis of patients undergoing FET for TAAD is one of the latest research objectives at our institution. The clinical case we report is part of a larger pilot study currently underway.

## Case presentation

### Methods

**Fig. 1:**
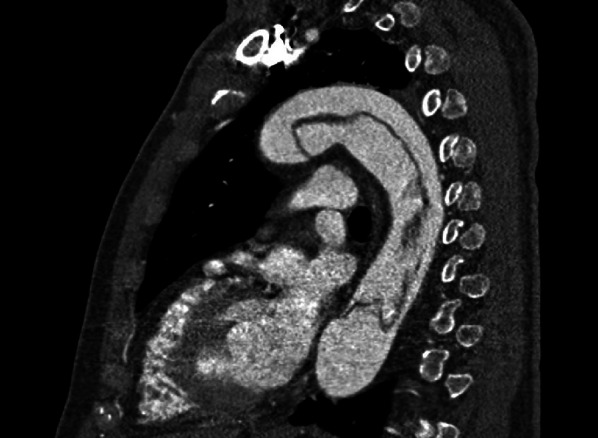
Preoperative sagittal-oblique CT angiography of the chest demonstrating acute type A aortic dissection with a primary intimal tear at the level of the aortic arch extending down in the thoracoabdominal aorta

We report a case of treating acute type A aortic dissection with the use of the Frozen Elephant Trunk technique in a JW patient. A 75-year old, 78-kg, hispanic man was admitted to our emergency room because abdominal and chest pain. His medical history included arterial hypertension and dyslipidemia. The computed tomographic (CT) scans showed an acute type A aortic dissection with a primary intimal tear at the level of the aortic arch extending down in the thoracoabdominal aorta (Fig. 1) The patient was candidated for emergency cardiac surgery. Preoperative echocardiography revealed tricuspid aortic valve with mild to moderate aortic insufficiency due to proximal aortic enlargement (type Ib according to the El Khoury classification). Admission laboratory data were Hb 15.2 g/dL, Hct 43,7%, and platelet count of 84.000/mmc. Autologous whole blood was collected in collection bags using a gravity-based method.

A median sternotomy was performed. Cardiopulmonary bypass (CPB), conducted in deep hypothermia (TC 20 °C), was instituted from right axillary artery through an interposed 8 mm Dacron graft (Gelweave™ Vascular graft, Terumo, UK) and the right atrium with a venous single two-stage cannula. Left ventricular venting achieved through the right superior pulmonary vein. At a temperature of 20 °C, CPB was discontinued, the supraortic branches clamped and bilateral antegrade cerebral perfusion performed. The stent-graft system (THORAFLEX HYBRID™ n°38, Terumo, UK) was introduced via anterograde in the true lumen of the descending aorta over a stiff guide-wire and released. When achieved optimum orientation and position the delivery system has been unsheathed. The distal anastomosis was performed between the incorporating sewing collar of the suture with external felt strip reinforcement in zone 1. The fourth branch of the graft was cannulated and the lower body perfusion was restored. Then the proximal anastomosis was performed at sinotubular junction by using a 4 − 0 polypropylene running suture with external felt strip reinforcement. The supraortic vessels were reimplanted to the side branches of the graft starting from the left common carotid artery, then brachiocephalic artery and finally the left subclavian artery through the interposed Dacron graft. After complete de-airing, systemic circulation to the brain was restarted from the side branch of the arch graft, while selective antegrade cerebral perfusion was stopped. At this point rewarming was initiated and weaning from CPB at nasopharyngeal temperature of 37 °C was unproblematic. All surgical field blood was saved and continuously reinfused with a cell saver. The correct opening of the stent-graft was controlled with transesophageal echocardiography (TEE). Accurate hemostasis was performed by means PerClot^®^ (CryoLife, Inc. U.S.A) technology and TISEEL^®^ (Baxter, Illinois, USA) fibrin sealant. The CPB time was 160 min, cross clamp time 63 min, selective antegrade cerebral perfusion time 85 min.

### Results

**Fig. 2:**
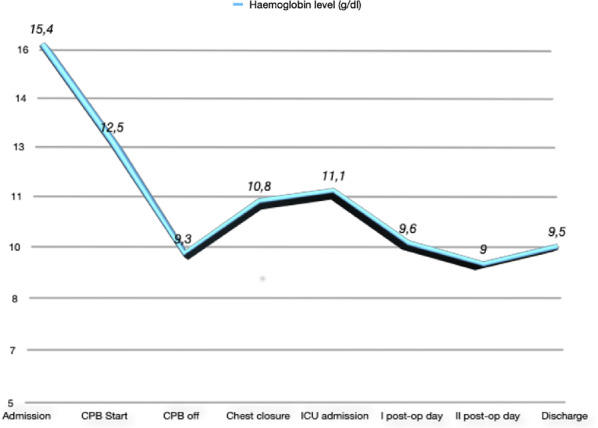
Trend of hemoglobin concentration during hospitalization

Hemoglobin concentration immediately after operation was 10.8 g/dl. We also examined the trend of hemoglobin values and this value can be seen during the entire operative phase and the subsequent hospitalization (admission, CPB start, CPB off, chest closure, intensive care unit (ICU) admission, post-operative (post-op) day, II post-op day and discharge) has never been below 9 g/dL (limit considered critical for the patient’s clinical and haemodynamic stability) (Fig. 2).

**Fig. 3:**
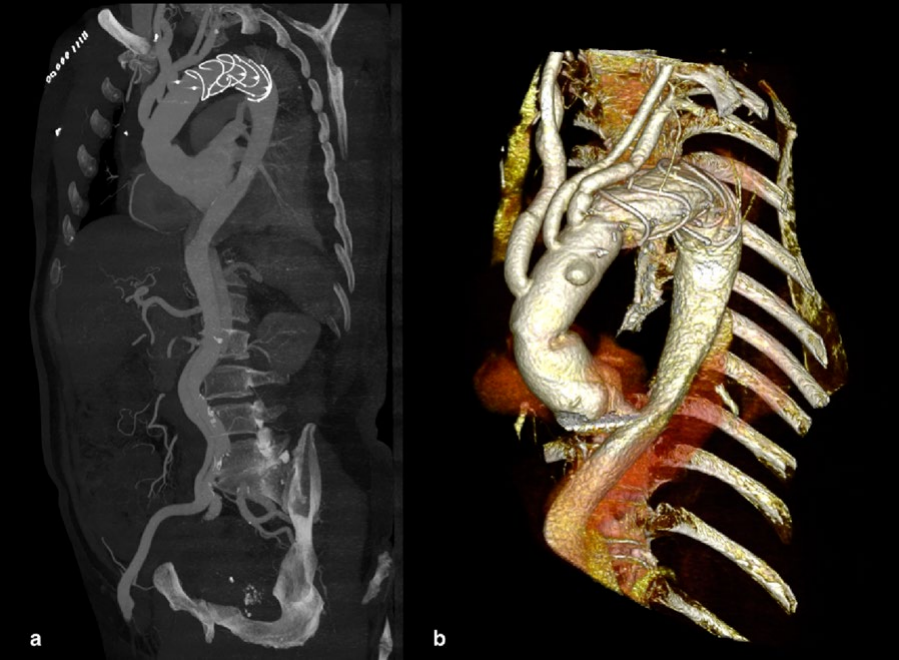
Postoperative CT. Maximum intensity projection (**a**) and three-dimensional CT (**b**) showing replacement of ascending aorta, aortic arch and FET in the descending aorta

The patient was transferred to the intensive care unit in stable condition without inotropic support. The patient’s recovery was regular. Two fase of atrial fibrillation was treated with Amiodarone successfully. The duration of mechanical ventilation support was 25 h. The duration of stay in the intensive care unit was 44 h. The postoperative hospital stay was 13 days. Postoperative Doppler echocardiography showed normal left ventricular function (LVEF > 60%) and mild aortic insufficiency. A postoperative CT scan was performed at discharge and revealed partial thrombosis of the false lumen and expansion of the true lumen of the descending thoracic aorta downstream of the prosthesis, without intratoracic collection and evidence of pseudoaneurysm (Fig. 3).

The patient was followed by our clinics through periodic checks which did not reveal any type of complication. Two and a half years after the operation, the patient underwent 4D Flow MRI analysis at our department.

Cardiac magnetic resonance (CMR) study was performed on a 3.0 Tesla Magnetom Prisma Siemens scanner (Siemens Healthineers AG, Erlangen, Germany). The patient was examined in the supine position, head first, using a respiratory sensor and electrocardiogram gating. The CMR protocol included baseline survey images, cines and 4D flow acquisition. For standard cines, we acquired 30 phases throughout the cardiac cycle. Other cine acquisition parameters include: Time to repeat (TR): 2.71, Time to echo (TE): 1.13, field of view (FOV): 360 × 289.3mm2 with phase FOV – 80.4%, number of signal averages (NSA): 1, matrix: 224 × 180 [Phase], bandwidth: 167.4 kHz, [930 Hz/Px], flip angle: 80, slice thickness: 8 mm and GRAPPA acceleration with a factor of 2. For 4D flow acquisition, the initial Velocity encoding (VENC) setting was 150–200 cm/s for all cases. We acquired 30 phases throughout the cardiac cycle to keep the data consistent with cines. The acquired temporal resolution was 40 ms. Other 4D flow acquisItions parameters include: TR: 4.98, TE: 2.71, field of view (FOV): 200 × 256.3 mm2, number of signal averages (NSA): 1, acquired voxel size = 2.5 × 2.5 × 2.5 mm3, bandwidth: 31.616 kHz, [494 Hz/Px], flip angle: 5, and GRAPPA acceleration in the phase-encoding direction with a factor of 2 and slide direction of 1. To avoid diastolic temporal blurring, the electrocardiogram was retrospectively gated with free breathing [6].

The acquired data were transferred to a postprocessing station and processed using Circle Cardiovascular Imaging software (v5.17, Circle Cardiovascular Imaging Inc, Alberta, Canada).

**Fig. 4:**
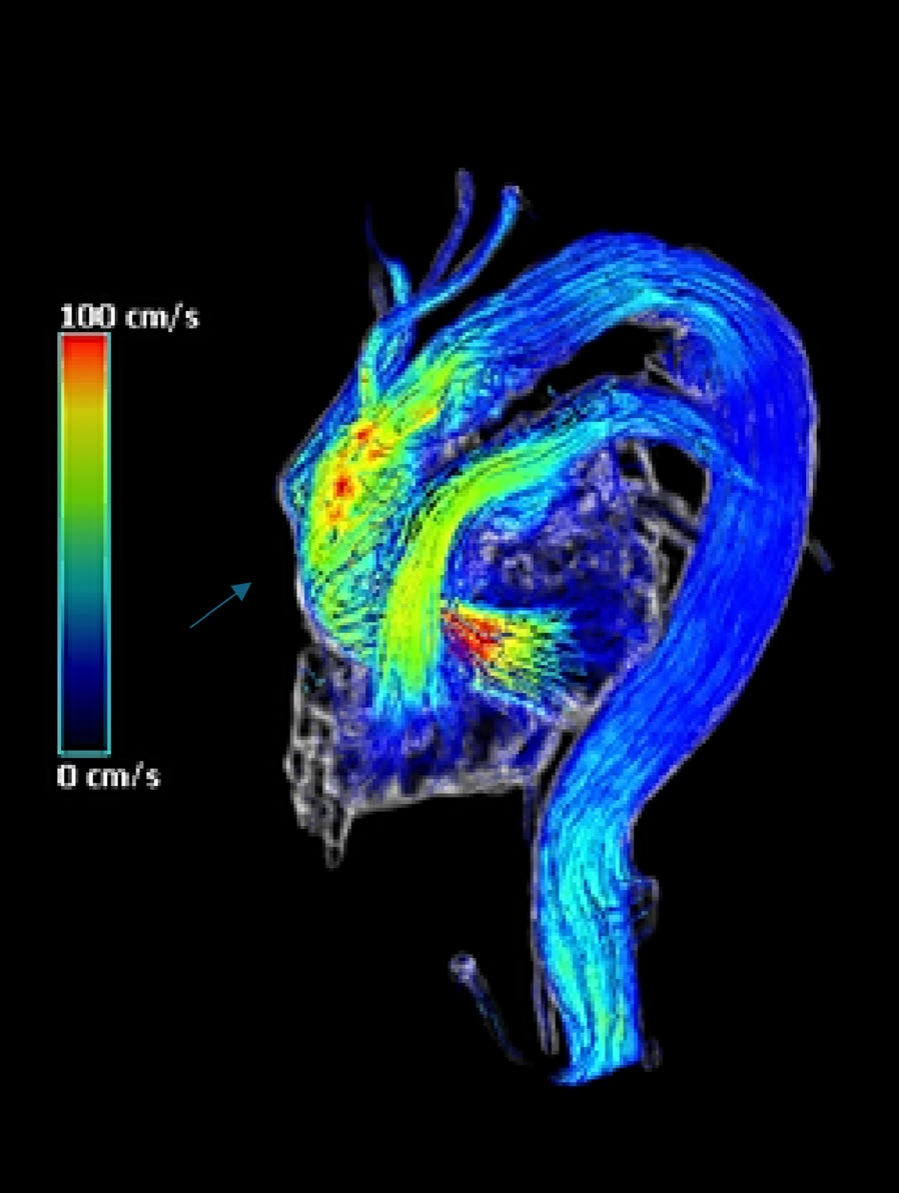
4D Flow MRI analysis: arrow: helical flow at the level of the ascending aorta, aortic arch and first portion of the aortic arch and progressive EL

The 4D Flow MRI analysis allowed us to study certain qualitative and quantitative parameters useful for evaluating the performance of the prosthesis used. In particular, in the qualitative evaluation of the case we report, the presence of an aberrant helical flow at the level of the aortic root, the ascending aorta and the first portion of the aortic arch was highlighted. By helical flow we meant the alignment with the rotation of the main flow. The quantitative evaluation instead allowed us to highlight a high degree of energy loss. Energy loss is defined as the loss of kinetic energy due to viscous friction against vessel walls and between blood layers. The EL can be highlighted by the progressive turning towards blue (lower values) of the velocity analyzed in the entire thoracic aorta (Fig. 4).

However, no vortex flows (rotation of the secondary flow around its axis) or other types of aberrant flows were highlighted. Therefore, the performance of the prosthesis used can be considered satisfactory. A single analysis is clearly not sufficient to reach scientifically valid considerations, therefore we believe it is appropriate to extend the 4D Flow MRI analysis to many more studies.

## Discussion

Perioperative bleeding is an important contributor to morbidity and mortality in cardiac surgery patients. The risk of hemorrhage is even greater in patients with type A dissections undergoing surgical repair. Thoracic aortic surgery in JW patients presents a unique challenge to the surgeon and anesthesiologist because the patient’s religious-based objection to the use of allogeneic blood products impacts surgical technique and intraoperative fluid management methodology. Repair of Stanford Type A aortic dissections without the use of blood products are possible and, though limited in number, reported with good outcomes in the literature [4, 7].

Given the exceedingly few cases in the JW population, the morbidity and mortality rate of operative management using a bloodless strategy remains unknown so many cardiovascular surgeons will decline to perform surgery under these circumstances. Thoracic aortic surgery in JW patients require extensive coordination from a multidisciplinary throughout the entire pre, intra and post-operative period. Optimizing hemoglobin values through the use of erythropoietin and IV iron is a major goal of preoperative management. In particular, it was seen that the use of erythropoietin in subjects who refused the transfusion was not associated with different outcomes compared to the control group [8]. The intraoperative surgical techniques used to maintain hemostasis include: minimize cardiopulmonary bypass and crossclamp time, minimize hemodilution, the use of intra-operative cell salvage, autologous blood trasfusion and the use of topical hemostatic agents. Paradoxically FET can reduce the risk of bleeding as it protects the distal anastomosis from bleeding by covering it with the stent thus facilitating the thrombotic obliteration of the false lumen. Efforts to prevent coagulopathy in the post-operative period were crucial and have been augmented with perioperative tranexamic acid, the use of clotting factors and intravenous iron to stimulate hematopoiesis. Furthermore, the increase in hemodynamic support can be useful to enhance tissue oxygenation [9].

From the implementation of these protocols it emerged that there are no substantial differences in terms of mortality in JW patients undergoing cardiac surgery. Similarly, morbidity in terms of acute myocardial infarction, stroke and infections was comparable.

However, the data relating to the onset of acute renal failure, length of hospitalization and reoperation for bleeding are still controversial [10].

Clearly, management cannot be optimized in patients undergoing emergency cardiac surgery and precisely in these cases it has been seen how severe anemia values (Hb < 8 g/dL) are associated with an increased rate of morbidity and mortality not only in patients who refuse the transfusion but also in those who receive only one single red blood cell transfusion [11]. Although, contrary to this, Vitolo and colleagues demonstrated that the perioperative outcome (myocardial infarction, re-exploration for bleeding) and the mortality rate of JW patients is comparable to the group of patients subjected to blood transfusions even in interventions performed in a state of emergency [12].

However, it is essential that both the patient and family members (especially if in emergency conditions which do not allow a dialogue with the interested subject) are adequately informed regarding the complications that could arise from cardiac surgery treatment without blood transfusion (ischemic events, neurological (even permanent) and in the most dramatic cases even death) [13]. This aspect takes on an even more important legal role in the case of minors.

In addition to the excellent surgical outcome of the operation and the absence of major complications in the peri- and post-operative period, we implemented our field of research with 4D Flow MRI analysis. This study allowed us to evaluate the performance of the prosthesis used through the study of qualitative and quantitative parameters.

4D Flow MRI analysis adds time as a fourth dimension, thus allowing a qualitative and quantitative study with the elaboration of flow models that allow a more accurate description of hemodynamics and the evaluation of fundamental parameters related to flows, such as wall shear stress, vortex and helical flows, distribution or EL [14].

Wall shear stress (WSS), i.e. the circumferential, axial and radial stresses due to variations in the pressure pulsation which are transferred to all layers of the aortic wall, is considered one of the main factors in the development and progression of aortopathy, as its values and its distribution are directly related to atherosclerosis, dysregulation of the extracellular matrix and degeneration of elastic fibers [15].

Helical and vortex flows have been considered pathological flow patterns. However, Kilner and colleagues showed the existence of a helical flow pattern towards the end of systole, leading to the preservation of laminar flow in the aortic arch. Therefore the helical flow in the case reported by us could be as similar as possible to the physiological flow. On the contrary, vortex flows are mostly associated with pathological conditions, such as aortic bicuspidity and aortic aneurysms, being in the latter case both cause and consequence [16]. Finally, EL is a quantitative parameter that is progressively gaining much importance. In fact, a high level of EL leads to two effects: on the one hand there is an excess amount of energy (lost) which is transferred to the vessel wall and on the other the loss of energy will cause an increase in afterload. Elevated EL values have been highlighted in patients suffering from thoracic aortic aneurysm and aortic stenosis [17].

What has been said so far leads us to the conclusion that 4D Flow MRI analysis can be a valid imaging technique not only in evaluating the performance of the prostheses used but also in the diagnosis and pre-operative follow-up of aortic aneurysms. In fact, thanks to the use of qualitative and quantitative parameters it allows us to analyze the evolution of the aneurysm process, in addition to the simple evaluation of the diameters. Furthermore, it is essential to remember how this test can be performed without any risk in young subjects (in reference to connective tissue pathologies: Marfan, Ehlers Danlos and Loeys-Dietz syndromes), pregnant women and patients with chronic renal failure with contraindication to the use of iodinated contrast medium.

## Conclusions

The case we report documents how very complex surgical interventions such as FET can be performed in JW patients in relative safety for the surgeon and the patient himself and adds to the limited number of reported cases of satisfactory repair of a massive aortic dissection using a bloodless strategy. It emerges from both our experience and the data in the literature that optimizing patient management has a crucial role in post-operative outcomes. Although the pre-operative protocol is not applicable in patients treated in an emergency state, however the strategies implemented intraoperatively (minimize cardiopulmonary bypass and crossclamp time, minimize hemodilution, use of intra-operative cell salvage, autologous blood transfusion and use of topical hemostatic agents) and the strengthening of post-operative management (increased haemodynamic support, use of clotting factors and intravenous iron) can have a positive impact on improving the outcome of JW patients. Furthermore, the 4D Flow MRI analysis made it possible to ascertain the stability of the aortic dissection, confirming the success of the operation and the excellent performance of the prosthesis used.

## Data Availability

The authors confirm that the data supporting the findings of this study are available within the article.
